# *Taylorella equigenitalis* in Icelandic intact males compared with other horse breeds using natural cover

**DOI:** 10.1111/evj.14121

**Published:** 2024-06-21

**Authors:** Markus Grabatin, Robert Fux, Yury Zablotski, Lutz S. Goehring, Tanja S. Witte

**Affiliations:** ^1^ Equine Clinic, Center for Clinical Veterinary Medicine, Faculty of Veterinary Medicine Ludwig‐Maximilians‐Universität München Munich Germany; ^2^ Division of Virology, Department of Veterinary Sciences Ludwig‐Maximilians‐Universität München Munich Germany; ^3^ MH Gluck Equine Research Center, College of Agriculture, Food and Environment University of Kentucky Lexington Kentucky USA

**Keywords:** contagious equine metritis (CEM), epidemiology, prevalence, stallion

## Abstract

**Background:**

Contagious equine metritis (CEM) is caused by *Taylorella equigenitalis*. It is a venereal disease that is detected in some breeds more than others and can cause temporary infertility with substantial costs for regular testing, sanitation and retesting. There was a perceived increase in *T. equigenitalis*‐positive cases in Icelandic intact males where natural cover is common.

**Objectives:**

We aimed to investigate the prevalence of *T. equigenitalis* in Icelandic intact males and compare to draught horse and Haflinger intact males. We hypothesised that prevalence of *T. equigenitalis* is higher in Icelandic compared with draught and Haflinger intact males.

**Study design:**

Cross sectional.

**Methods:**

Swabs from 76 Icelandic, 35 Haflinger, and 51 draught horse intact males were collected on 38 different farms and analysed by qPCR. Animals were further stratified into active breeding and non‐breeding animals and age groups (1.5–7.0 and 8.0–26.0 years). Fisher's exact tests and mixed effect logistic regression with ‘farm’ as random effect were used to estimate differences in odds for T. *equigenitalis*‐positive test results.

**Results:**

The overall prevalence of *T. equigenitalis* in included intact males was 16.7% (27/162). The odds for *T. equigenitalis*‐positive intact males were significantly higher in Icelandic compared with draught and Haflinger intact males (Odds ratio [OR] = 6.42, 95% confidence interval (CI) = 1.43–28.8, *p* = 0.02). Odds for *T. equigenitalis*‐positive intact males were significantly lower in active breeding compared with non‐breeding animals (OR = 0.09, 95% CI = 0.01–0.54, *p* = 0.009). Age had no significant influence on test results.

**Main limitations:**

Convenience sampling with regional restrictions to Southern Germany and Austria, small sample size.

**Conclusions:**

Significantly higher odds for *T. equigenitalis‐*positive intact males were found within Icelandic over draught and Haflinger and within non‐breeding animals compared with active breeding animals. Findings suggest that non‐breeding animals could be a reservoir for *T. equigenitalis*. Testing for CEM should therefore be routinely performed in Icelandic horses prior to breeding and investigations into epidemiology and reservoirs on affected farms should be initiated.

## 1 INTRODUCTION

Contagious equine metritis (CEM) is a widespread equine disease with recurring outbreaks worldwide since its first description in 1977.^1^, ^2^ The causative agent of CEM is *Taylorella equigenitalis*, a gram negative, non‐motile coccobacillus.^3^, ^4^ The transmission is usually venereal, but can also be iatrogenic due to poor breeding hygiene.^2^ Foals born to infected mares are thought to become chronic carriers,^5^, ^6^ and fomite transmission may occur.^1^, ^5^ However, persistence of the organism in the environment over time has not been proven.^5^, ^6^ Stallions can be subclinical carriers of *T. equigenitalis* on their external genitalia,^1^, ^7^ while in mares, a clinical infection might cause severe inflammation of the reproductive tract, associated with short‐term infertility.^8^ Clinical signs in mares differ and range from mild to severe endometritis, cervicitis, and vaginitis with purulent vaginal discharge.^2^, ^8^ In rare cases, *T. equigenitalis* might cause abortion.^9^ However, the course of disease can also be subclinical with a chronic and persistent subclinical carrier state.^8^ Pre‐breeding sampling is commonly conducted in stallions and mares as recommended by Horserace Betting Levy Board (HBLB) International Codes of Practice.^10^ Testing is currently performed via culture or PCR assay from swabs collected from urethral fossa, urethra, and penile sheath in stallions and from clitoral fossa and sinuses in mares.^11^, ^12^

Infections or outbreaks can cause high economic losses due to reduced pregnancy rates, labour‐intensive treatments and enforced sexual rest. This was especially true for the Thoroughbred breeding industry during large outbreaks in 1977 and 1978 (United Kingdom, Ireland and United States).^13^, ^14^ Because of mandatory natural cover and low stallion‐to‐mares ratio, Thoroughbred breeding associations worldwide have implemented strict testing rules within the breed (including ‘teaser’ stallions and mares).^14^, ^15^ With this regime, the manifestation of the classical venereal transmitted disease has been nearly eradicated from the global Thoroughbred population.^1^, ^16^ However, CEM has been reported in various other breeds worldwide including Europe and was assumed to be endemic in some non‐Thoroughbred populations.^1^, ^14^ *Taylorella equigenitalis* isolates from Germany have been held responsible for CEM outbreaks in the United States.^17^ Within the European Union (EU), *T. equigenitalis* transmission via fresh or frozen semen in any breed is prevented by rigorous CEM testing before semen collection from breeding stallions according to EU regulations.^12^ However, naturally covering stallions are exempt from testing. Sporadic cases of *T. equigenitalis*‐positive horses have been reported in Germany in recent years.^18^

While pasture breeding without mandatory and standardised *T. equigenitalis* testing is common among Icelandic horses, there has also been a perceived increase in *T. equigenitalis*‐positive cases and clinical impact of CEM within this breed. Furthermore, Denmark reported a major outbreak among Icelandic horses in 2020 raising awareness of the disease in this breed.^19^

Aim of this study was to investigate the prevalence of *T. equigenitalis* in Icelandic intact males (Icelandic) and compare findings to a comparison group of intact male Haflinger and draught horses (draught and Haflinger). We chose to compare these breeds because their mares are usually covered at pasture or in‐hand by natural cover and neither breeding association specifies mandatory testing prior to breeding. We hypothesised a higher prevalence of *T. equigenitalis* in Icelandic over draught and Haflinger. To the author's knowledge, this is the first investigation into *T. equigenitalis* prevalence in these breeds in a region spanning Southern Germany and parts of Austria.

## 2 MATERIALS AND METHODS

### 2.1 Animals

Between January 2020 and March 2021, we collected samples from a total of 162 intact male horses on 38 individual farms (76 Icelandic on 10 farms, and 86 draught and Haflinger on 28 farms, see Table 1). Study participants were acquired by contacting owners of intact males out of the Equine Clinic (LMU Munich) records pool or approaching animal owners of hospitalised animals following a convenience sampling strategy. Horses were included by breed (Icelandic horses, Haflinger, draught horses) and sex (intact male). It was not always possible to test every intact male on each farm due to different ownership. Most farms were in a 200 km radius around Munich (Germany), which includes parts of Austria. Only a single animal was tested from central Germany which was a referred case to the Equine clinic (LMU Munich) for purposes unrelated to male reproduction. We obtained a breeding history and age of all included intact males prior to sampling. Additional information was obtained via a retrospective telephone survey 2 years later. Information on husbandry, herd health, infectious diseases and treatments prior to testing were obtained (see Questionnaire S1).

**TABLE 1:** Total numbers of intact males in individual groups and total numbers of included farms.

	Icelandic intact male (*n* = 76)			COM (*n* = 86)		
	Total	Positive	Negative	Total (draught horse intact male/Haflinger intact male)	Positive (draught horse intact male/Haflinger intact male)	Negative (draught horse intact male/Haflinger intact male)
Total	76	23	53	86 (51/35)	4 (4/0)	82 (47/35)
Active breeding animal	28	3	25	54 (24/30)	0 (0/0)	54 (24/30)
Non‐breeding animal	48	20	28	32 (27/5)	4 (4/0)	28 (23/5)
Farms	10	4	6	28 (14/12/2*)	2 (2/0)	26 (12/12/2*)

*Note*: A farm was defined positive as soon as one intact male was tested *T. equigenitalis*‐positive. The asterisk (*) indicates farms where both, Haflinger and draught horse intact males, were tested.

Icelandic intact males were compared with draught and Haflinger, a combined group of draught horse and Haflinger intact males. In both groups, intact males were further divided into active (or previously used for) breeding (ABA), and non‐breeding (never have been, or not yet) animals (NBA) as well as two age groups of 1.5–7.0 and 8.0–26.0 years.

### 2.2 Sampling procedure

In most cases, sample collection was performed at the home farm. In 13 cases (Icelandic *n* = 3, draught horses *n* = 8, Haflinger *n* = 2), sample collection was performed at the Equine clinic (LMU Munich) because of a hospital stay unrelated to male reproduction. Preferably, intact males were stimulated by a mare for penile let down. If that was not possible, they were sedated orally with detomidine hydrochloride (Domosedan‐Gel, Orion Corporation) to induce penile extrusion. Samples were collected from the urethral fossa, urethra, and penile sheath with a sterile polyester swab (Copan Italia S.p.A.) as described by EU regulation EU2020/686 and by the HBLB International Codes of Practice.^10^, ^12^ Swabs from the three locations were stored separately in 1.5 mL tubes (Eppendorf SE) containing 400 μL NaCl 0.9% (Ecotainer, B. Braun Melsungen AG). Samples were immediately put on ice, transported to the laboratory of the Equine Clinic and stored there at −80°C until further investigation.

### 2.3 DNA extraction and quantitative PCR

A volume of 25 μL from each sample per animal was combined for pooled DNA extraction using the DNeasy Blood and Tissue Kit (Qiagen) according to the manufacturer's instruction.^20^ For quantitative PCR (qPCR), the SensiFAST Probe Lo‐ROX Kit (Meridian Bioscience) was used. A slightly modified qPCR protocol for *T. equigenitalis* detection previously described by Wakeley et al. was used.^21^ The thermal profile of the qPCR was set at: 95°C for 5 min, and 42 cycles of 94°C for 15 s and 60°C for 60 s. AriaMx real‐time qPCR system (Agilent) and its Aria 1.8 software were used for analysis. Negative and positive controls were added to each run. Results with a Ct‐value of <35.0 were regarded as positive samples.

### 2.4 Data analysis

Statistical analyses were conducted using R version 4.3.1. (The R Foundation for Statistical Computing). Additionally, mean, median, standard deviation (SD) and interquartile range (IQR) values as well as percentages were calculated using Microsoft® Excel for Mac (Version 16.76, 2023).

The age of all included animals was investigated for normal distribution using the Shapiro–Wilk test. The relationship between *T. equigenitalis‐*positivity and ‘breed’ as well as ‘breeding use’ was assessed using the univariate Fisher's exact test (fisher. test stats version 3.6.2.). Differences in prevalence of *T. equigenitalis*‐positive intact males were further evaluated by multivariable mixed effect logistic regression with ‘farm’ as random effect (glmer (lme4_1.1‐35.1)). The odds for a *T. equigenitalis*‐positive qPCR result among intact males were assessed by estimated marginal means. Two age groups were used in the logistic regression analyses. The age groups were as evenly distributed as possible and contained at least one positive animal in each group (1.5–7.0 years and 8.0–26.0 years, respectively). Results with a *p*‐value <0.05 were considered statistically significant.

## 3 RESULTS

### 3.1 Animals

In total, 162 intact males between 1.5 and 26 years (median: 5.0 years; IQR: 6.0 (3.0–9.0) years) were included in this study. Penile swab samples from all 3 locations were collected from all tested intact males. There were slightly more draught and Haflinger (53.1%, 86/162) than Icelandic horses (46.9%, 76/162) and within the comparison group, there were 41% (35/86) Haflinger, and 59% (51/86) draught horse intact males (Table 1). Overall, 51% (82/162) actively breeding and 49% (80/162) non‐active intact males were tested (Table 1).

The age of tested intact males was not normally distributed (*p* < 0.001). The number of intact males in each age group was similar between breed groups (Icelandic 1.5–7.0 years, *n* = 56; draught and Haflinger 1.5–7.0 years, *n* = 56; Icelandic 8.0–26.0 years, *n* = 20; draught and Haflinger 8.0–26.0 years, *n* = 30; see Table 2). A higher number of non‐active intact males was found between 1.5 and 7.0 years (non‐active *n* = 73, active 39), in contrast to intact males between 8.0 and 26.0 years (non‐active *n* = 7, active *n* = 43).

**TABLE 2:** Numbers of *T. equigenitalis*‐positive intact males and total numbers of all included intact males in both age groups divided into Icelandic intact male, comparison group, active breeding animal and non‐breeding animal.

	Age: 1.5–7.0 years		Age: 8.0–26.0 years	
	Active breeding animal	Non‐breeding animal	Active breeding animal	Non‐breeding animal
Icelandic intact male	3/9	19/47	0/19	1/1
Comparison group	0/30	1/26	0/24	3/6
Total	3/39	20/73	0/43	4/7

Number of tested intact males per farm varied: Icelandic stallions were distributed over 10 different farms with an age distribution from 1.5 to 26.0 years (median: 4.0 years; IQR: 5.0 (3.0–8.0) years). Draft and Haflinger intact males were distributed on 28 different farms with an age distribution of 2.0–18.0 years (median: 6.0 years; IQR: 6.8 (3.0–9.8) years). In general, breeds were housed separately on individual farms except on two farms where draught horses and Haflinger intact males were housed together. In the Icelandic group, 37% (28/76) of the intact males were actively breeding and in draught and Haflinger, 63% (54/86) of the intact males were actively breeding (Table 1).

Additional farm information that could not be obtained during the sample collection visit was obtained by retrospective questionnaire in November 2023; however, only 63% (24/38) of the contacted owners responded. All Icelandic farms (*n* = 7) stated that they used group housing; however, 6 of 7 Icelandic farms also reported using single boxes with access to pasture. All draught and Haflinger farms (*n* = 17) reported single box housing with access to pasture. Four of 17 draught and Haflinger farms had additional group housing. Prior to or at the time of sampling, none of the included farms reported an infectious disease outbreak or consecutive treatment with antibiotics of a large proportion of the farm population. Therefore, we assume that antibiotic treatment did not influence our results. Further results of the telephone survey are presented as Table S1.

### 3.2 Quantitative PCR results

#### 3.2.1 Quantitative PCR results regarding breed

Overall, *T. equigenitalis* could be detected in 16.7% (27/162) of all tested intact males (*n* = 162) included in the study with Ct‐values between 14.77 and 34.04 (median = 18.54, IQR = 5.72). Within the Icelandic group (*n* = 76), 30% (23/76) and within the draught and Haflinger group (*n* = 86) 5% (4/86) were *T. equigenitalis*‐positive; however, *T. equigenitalis*‐positive qPCR results were exclusively detected in draught horse intact males (Table 1).

The odds for *T. equigenitalis*‐positive intact males were significantly increased for Icelandic compared with draught and Haflinger (Fischer's exact, Icelandic vs. draught and Haflinger odds ratio (OR) = 8.78, 95% confidence interval (CI): 2.78–36, *p* < 0.001). Separating draught and Haflinger into Haflinger and draught horse intact males, significantly higher odds were detected in Icelandic compared with draught horse intact males only (Fischer's exact, Icelandic vs. draught OR = 5.04, 95% CI: 1.56–21.5, *p* = 0.004). When we excluded one Icelandic farm (I1) because it had a high prevalence (15 positives of 34 intact males), the odds for *T. equigenitalis*‐positive intact males were still higher for Icelandic compared with draught and Haflinger (Fischer's exact, Icelandic without farm I1 vs. draught and Haflinger OR = 4.75, 95% CI: 1.18–23.06, *p* = 0.02). Analysing the odds for *T. equigenitalis*‐positive intact males in Icelandic compared with draught and Haflinger using logistic regression taking ‘farm’ as random effect, significantly higher odds for *T. equigenitalis*‐positive intact males were found in Icelandic compared with draught and Haflinger (logistic regression Icelandic vs. draught and Haflinger OR = 6.42, 95% CI: 1.43–28.8, *p* = 0.02).

#### 3.2.2 Quantitative PCR results regarding breeding use

Within the Icelandic group, 11% (3/28) active breeding animals and 42% (20/48) non‐breeding animals had a *T. equigenitalis*‐positive qPCR result. In draught and Haflinger group, 13% (4/32) *T. equigenitalis*‐positive intact males were detected in the group of non‐breeding animals and none in the group of active breeding animals. Combining tested intact males of all breeds, the odds for *T. equigenitalis*‐positive intact males were significantly lower in actively breeding compared with non‐active males (Fischer's exact active vs. non‐active OR = 0.09, 95% CI: 0.02–0.32, *p* < 0.001). Using logistic regression with ‘farm’ as random effect, the odds for *T. equigenitalis*‐positive intact males were also lower in actively breeding compared with non‐active animals (logistic regression active vs. non‐active OR = 0.09, CI: 0.01–0.54, *p* = 0.009).

#### 3.2.3 Quantitative PCR results regarding age

**FIGURE 1:**
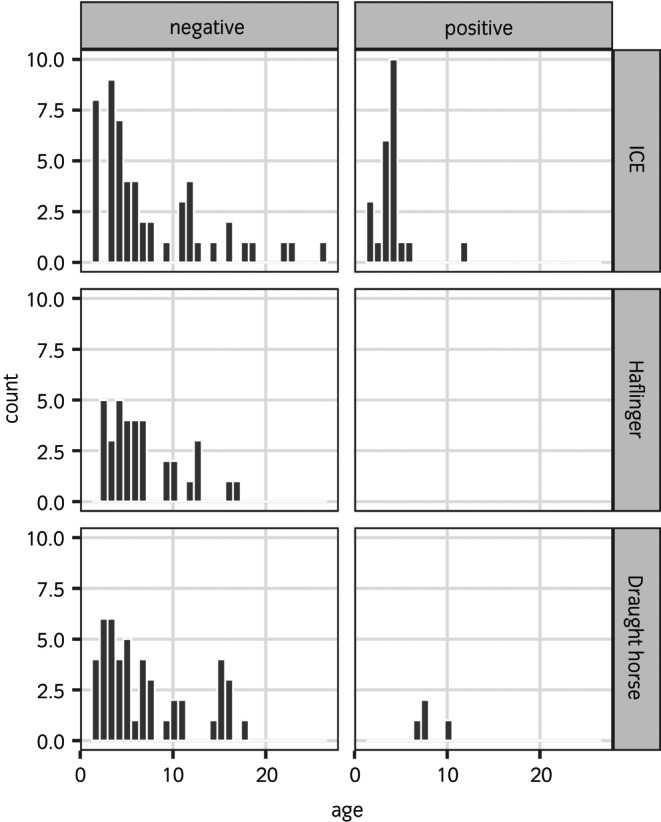
Age distributions in Icelandic, Haflinger and draught horse intact males differentiated according to *T. equigenitalis*‐positive and negative qPCR results. Draught horse, draught horse intact male; Haflinger, Haflinger intact male; ICE, Icelandic intact male; negative, *Taylorella equigenitalis*‐negative intact males; positive, *Taylorella equigenitalis*‐positive intact males.

The age distribution of all *T. equigenitalis*‐positive intact males was between 1.5 and 12.0 years (median: 4.0 years; IQR: 1.5 (3.0–4.5) years). The age distribution in the different breeds split into intact males with *T. equigenitalis*‐positive and *T. equigenitalis*‐negative qPCR results is shown in Figure 1. No significant difference in odds for a *T. equigenitalis*‐positive intact male was detected between the age groups using logistic regression with ‘farm’ as random effect (logistic regression 1.5–7.0 years vs. 8.0–26.0 years OR = 0.51, 95% CI: 0.09–2.83, *p* = 0.4).

#### 3.2.4 Quantitative PCR results regarding farm

All intact males were distributed over 38 different and independent farms (Table 1). In Icelandic, 40% (4/10) and in draught and Haflinger, 7% (2/28) of the farms were detected with at least one *T. equigenitalis*‐positive intact male and the farm was therefore regarded as positive. In *T. equigenitalis*‐positive Icelandic farms, one farm housed 45% (34/76) of all tested Icelandic and 65% (15/23) of all *T. equigenitalis*‐positive Icelandic. In two draught and Haflinger farms, both, draught horse and Haflinger intact males, were housed and tested. In these farms, all tested intact males were *T. equigenitalis*‐negative (Table 1).

## 4 DISCUSSION

This study shows increased risk of *T. equigenitalis‐*positive in Icelandic compared with draught and Haflinger intact males. We compared Icelandic to a combined group of Haflinger and draught horse intact males because of similar breeding requirements using mostly natural cover with no mandatory testing. Higher odds for *T. equigenitalis‐*positive intact males were also shown in non‐breeding compared with actively breeding animals, regardless of breed.

According to the information we received from farms answering the retrospective telephone survey, 100% of the Icelandic farms and only 24% of draught and Haflinger farms used group housing. This corresponds to common practices in housing of breeding animals and bachelor stallions in Iceland and typical housing conditions of Icelandic horses in European Nordic countries, where extensive group housing is the predominant husbandry form.^22^, ^23^ Breeding practice in Icelandic horses often involves pasture breeding where a group of mares is combined with a stallion for several weeks during breeding season. This contrasts with Haflinger and draught horses, where in hand breeding is more common. Furthermore, it is not uncommon for Icelandic horses to have yearling horses on pasture as bachelor herds accompanied by one of the older stallions.

Initial analysis showed significantly higher odds for *T. equigenitalis*‐positive in Icelandic compared with draught and Haflinger intact males. One of the 10 included Icelandic farms (I1) housed 45% (34/76) of all Icelandic animals and 65% (15/23) of the *T. equigenitalis*‐positive Icelandic animals found in this study. This stable performed regular *T. equigenitalis* tests and had already recognised positive cases prior to this study. However, excluding this farm from the analysis, also showed the odds of positive results in Icelandic intact males were higher compared with draught and Haflinger. In total, *T. equigenitalis*‐positive intact males were detected on 6 farms. On 5 of these farms between 40% and 50% of the intact males were *T. equigenitalis*‐positive. These results suggested a possible farm effect due to cross infection or other influencing factors between tested horses on one farm. Therefore, a farm had to be regarded as a cluster and the clustering effect was taken into account using logistic regression with ‘farm’ as a random effect and with this analysis method, odds for *T. equigenitalis*‐positive intact males remained higher for Icelandic compared with draught and Haflinger animals. Our finding of an increased prevalence of *T. equigenitalis* among tested Icelandic is supported by observations made during a major CEM outbreak in Denmark reported by the Danish Statens Serum Institute in 2020: in 700 samples from 269 horses of different breeds, 104 Icelandic horses (14 intact males and approximately 90 mares) were found *T. equigenitalis*‐positive.^19^ In the Netherlands, *T. equigenitalis*‐positive horses of various breed and age (6/13 maiden mares, 48/94 bred mares), were detected by Parlevliet et al. in 1995.^24^ During their investigation of these 107 mares with no clinical signs of CEM, 49% were *T. equigenitalis*‐positive in a PCR‐assay. Moreover, in the same study, 29.2% (7/24) of mares imported from Iceland were found to be *T. equigenitalis*‐positive before contact to horses in continental Europe, suggesting that *T. equigenitalis* has been present within the Icelandic horse population in Iceland without apparent clinical signs or detectionn.^24^ The statement that CEM may be an endemic disease in central Europe has been made by other observers studying other non‐Thoroughbred breeds.^1^, ^14^, ^25^

In our study, the group of Haflinger intact males stands out as there were no *T. equigenitalis*‐positive test results. This finding should be regarded with caution as it could be due to the convenience sampling method and study design we used and this is an important limitation which could have introduced bias across the whole study population. A further limitation is our deviation from test recommendations: The HBLB code of practice for breeders recommends taking additional samples of pre‐ejaculate fluid if possible^10^ and this was not performed in the current study. Furthermore, the included stallions were sampled only once and therefore, the testing methods did not comply with the regulations according to EU law or HBLB recommendations, where two samples at intervals of 7 days are prescribed.^10^, ^12^ Due to this deviation from the official recommendations, it is conceivable, albeit unlikely, that a number of intact males were false negatives in our study. Nevertheless, the single sample approach used in the current study did not differ between groups and therefore, any error should occur to a similar extent in all included horses.

Another reason that Haflinger intact males were *T. equigenitalis*‐negative might be there was a high number of actively breeding animals within this group. In general, to avoid breeding failure by CEM, it can be assumed that actively breeding animals are tested more often and more closely monitored than non‐breeding animals. However, we obtained in feedback from 71% (10/14) of the farms housing Haflinger intact males and none of these stables had carried out CEM tests prior to our study (see Table S1). Another possible explanation for a lack of *T. equigenitalis*‐positive Haflinger intact males might be the low number of non‐breeding Haflingers which were tested (*n* = 5) compared with other breeds (Icelandic non‐breeding: *n* = 48, draught horse non‐breeding: *n* = 27). All (4/4) of the *T. equigenitalis*‐positive draught horse intact males and 87% (20/23) of the positive Icelandics were found in non‐breeding animals. However, significantly higher odds for *T. equigenitalis*‐positive intact males in Icelandic compared with only draught horse intact males was also shown albeit with large confidence intervals due to the small number of tested horses.

A further notable result is the significantly higher prevalence of *T. equigenitalis*‐positive results in non‐breeding compared with actively breeding. This observation was unexpected given that natural cover is the main source of infection. Therefore, other routes of transmission must be taken into consideration. Intrauterine or periparturient infection of foals born to infected mares were proposed by Timoney and Powel based on the history of positive colts and fillies and their parents.^5^ In our cases, this route of transmission might be possible because we detected *T. equigenitalis* in very young Icelandics never used for breeding. However, age had no influence on the odds of *T. equigenitalis*‐positive test results in our study (Figure 1). Sampling of foals and dams immediately after birth until the age of yearlings would be necessary to provide more information regarding a possible intrauterine or periparturient infection. Iatrogenic transmission or poor hygiene standards at semen collection can play a role in transmission.^2^, ^26^ However, that route of transmission is likely to be negligible in non‐breeding animals in the current study due to absence of breeding related activities. As extensive group housing is common on Icelandic horse farms, environmental transmission or transmission from direct contact in group housing cannot be ruled out. However, until now, there is no evidence for persistence of *T. equigenitalis* in an environmental reservoir for an extended time or its transmission afterwards.^6^ Allombert et al.^27^ proved that *T. equigenitalis* is able to survive under laboratory conditions for at least 7 days in an amoeba (*Acanthamoeba castellanii*) and proposed this as a possible mechanism for *T. equigenitalis* to survive in the environment.^27^ There is a need for further investigations and knowledge on possible further ways of transmission and environmental sampling to help in understanding and preventing transmission.

In Germany, *T. equigenitalis* is a legally notifiable pathogen.^28^, ^29^ Between 2018 and 2022, increased numbers of annual infections were documented compared with the previous years with up to 61 animals (mean: 41.4) officially reported as *T. equigenitalis*‐positive per year in Germany.^18^ We assume that officially reported *T. equigenitalis*‐positive cases are predominantly breeding animals, because subclinical carriers not used for breeding are commonly not tested. Therefore, based on our observations of relatively high numbers of *T. equigenitalis*‐positive in non‐actively breeding animals, the number of *T. equigenitalis*‐positive horses may be greater than the officially reported numbers. It is likely that the current testing and treatment strategies and notification requirements are not sufficient to prevent further outbreaks.

In the current study, a high number of *T. equigenitalis*‐positive intact males of younger age in Icelandic was noted (Figure 1) but no statistically significant influence of age on odds for a *T. equigenitalis‐*positive qPCR result was shown in this relatively small study. Nonetheless, our data suggest that other non‐venereal transmission routes as proposed by Timoney and Powell,^5^ might exist. In Icelandic farms, bachelor herds of yearlings are often accompanied by one of the older stallions. Contact infection between those intact males due to physical contact between genital or even nasal mucosa and a possible environmental contamination, which has not been described for *T. equigenitalis* yet, should be explored further.^6^

A main limitation of this study was the convenience sampling due to testing participants through a call for study participation and patients of the Equine Clinic (LMU Munich). This led to bias regarding the study population and limits the ability to draw a conclusion on the total Icelandic horse population. Further studies examining a representative sample of an entire local population are necessary to definitively determine the prevalence of *T. equigenitalis* in the investigated horse breeds. Another limitation was insufficient information about the relationships between intact males. We attempted to address this through additional information gathering by a retrospective telephone survey. However, a failure to collect complete data from all farms prevented this, which might be due to the time lag of 2 years between sampling and survey. Therefore, further studies investigating the total population of exposed farms together with investigation of the CEM status of newborn foals and their dams could gain further insights into other routes of transmission. Studies examining different *T. equigenitalis* strains in the investigated breeds could help to give a better understanding about differences on DNA‐level of *T. equigenitalis* in different breeds. Of 124 field isolates from Germany and Austria, Sting et al. found the majority of *T. equigenitalis* isolates in Lipizzaner, Icelandic horses and draught horses, whereupon the Icelandic isolates differed partially from those of other breeds.^25^ Bleumink‐Pluym et al. could show differences between strains in the ability to invade and replicate in equine cells in culture. Therefore, they suggested a different virulence between those *T. equigenitalis* strains.^30^ Further studies investigating the underlying strains in different breeds would be of great interest as proposed by Sting et al.^25^ Evaluation of fertility according to *T. equigenitalis* strains and horse breeds could provide further information.

Breeding by natural cover in any breed should only occur if appropriate testing has been carried out. Additionally, our results suggest *T. equigenitalis* should also be monitored in non‐breeding animals which have contact with breeding animals. Within the current EU regulations, natural cover is exempt from testing and eradication of CEM is therefore not possible in all breeds.^12^ In order to gain a CEM‐free status, a control system compatible with the HBLB codes of practice for breeders should be established for all breeds using natural cover.^10^ However, within the Icelandic horse population, this is likely to be hard to achieve for economic reasons.

## 5 CONCLUSION

The prevalence of *T. equigenitalis* was investigated in breeds using mainly natural cover. The pathogen responsible for CEM was detected in Icelandic horses with higher odds compared with Haflinger and draught horse intact males. Although the venereal transmission is generally thought to be the main source of infection, we found the odds of a *T*. *equigenitalis*‐positive result was significantly higher in non‐actively breeding compared with actively breeding animals. Testing and outbreak control for *T. equigenitalis* should therefore always be considered in breeding horses regardless of the breeding method used. Further studies analysing the genotype of the underlying bacterial strain as well as sampling the whole population of affected farms including their environment could help to gather more epidemiological data and to better understand possible transmission routes.

## FUNDING INFORMATION

Partially funded from the German Equine Veterinary Association/Deutsche Gesellschaft für Pferdemedizin (GPM).

## CONFLICT OF INTEREST STATEMENT

The authors declare no conflicts of interest.

## AUTHOR CONTRIBUTIONS

**Markus Grabatin:** Conceptualization; data curation; formal analysis; writing – original draft. **Robert Fux:** Formal analysis; methodology; project administration. **Yury Zablotski:** Formal analysis; methodology. **Lutz S. Goehring:** Conceptualization; methodology; writing – review and editing. **Tanja S. Witte:** Conceptualization; formal analysis; investigation; project administration; supervision; writing – original draft; writing – review and editing.

## DATA INTEGRITY STATEMENT

T. S. Witte had full access to all the data in the study and takes responsibility for the integrity of the data and the accuracy of data analysis.

## ETHICAL ANIMAL RESEARCH

The study was approved by the ethics committee of the Center of Clinical Veterinary Medicine, Faculty of Veterinary Medicine of the Ludwig‐Maximilians University München, Germany (Reference number 192‐11‐11‐2019).

## INFORMED CONSENT

The consent for the investigations was given by the horse owners or their agents.

### PEER REVIEW

The peer review history for this article is available at https://www.webofscience.com/api/gateway/wos/peer‐review/10.1111/evj.14121.

## Supporting information


**Questionnaire S1.** Questionnaire home stables (telephone call).

**Table S1.** Overview of the farms housing the horses included in the study.

## Data Availability

The data that support findings of this study are available from the corresponding author upon reasonable request: Open sharing exemption granted by the editor.
