# Supplementary material for: Taylorella equigenitalis in Icelandic intact males compared with other horse breeds using natural cover
Source: Equine Vet J. 2024 Jun 21;57(2):441–8. doi: 10.1111/evj.14121 (PMC11807930; doi:10.1111/evj.14121)
Supplement: Supplementary file 1 — Questionnaire S1. Questionnaire home stables (telephone call). [file EVJ-57-441-s001.pdf]

Equine Veterinary Journal BEVA

Questionnaire S1:

Questionnaire home stables (telephone call)

Type of farm: ☐: breeding farms / ☐: riding farm / ☐: other farms

Number of horses / Equids:

☐: < 50 horses / ☐: 50-100 horses / ☐: > 100 horses

Number of stallions:

☐: < 5 stallions / ☐: 5-20 stallion / ☐: > 20 stallion

Number of mares:

☐: < 5 mares / ☐: 5-20 mares / ☐: > 20 mares

Number of geldings:

☐: < 5 geldings / ☐: 5-20 geldings / ☐: > 20 geldings

Horse breeds:

☐: Icelandic / ☐: Haflinger / ☐: Draft horse / ☐: other breeds

Contact between horse breeds:

☐: Yes / ☐: No

Husbandry: ☐: box housing with pasture / ☐: group housing

Contact between the stallions ☐: Yes / ☐: No (☐direct/ ☐indirect)*

Contact between stallions and geldings ☐: Yes / ☐: No (☐direct/ ☐indirect)*

Contact between stallions and mares ☐: Yes / ☐: No (☐direct/ ☐indirect /☐ breeding)*

CEM outbreaks at the farm:

☐: Yes / ☐: No (☐: 1 / ☐: 1-3 / ☐: >3)

Treatment of CEM:

☐: Yes / ☐: No (☐: Every time / ☐: Differently / ☐: None)

Testing for CEM routinely:

☐: Yes / ☐: No

Other disease outbreaks? ☐: Yes / ☐: No

Treatment: ☐: Yes / ☐: No

** direct contact: The animals had physical contact with each other; indirect contact: The animals used the same stables, pastures or facilities at different times.*

.
