# Supplementary material for: Taylorella equigenitalis in Icelandic intact males compared with other horse breeds using natural cover
Source: Equine Vet J. 2024 Jun 21;57(2):441–8. doi: 10.1111/evj.14121 (PMC11807930; doi:10.1111/evj.14121)
Supplement: Supplementary file 2 — Table S1. Overview of the farms housing the horses included in the study. [file EVJ-57-441-s002.pdf]

Equine Veterinary Journal BEVA

Table S1:

Overview of the farms housing ICE (Icelandic intact males), HAF (Haflinger intact males), Draught (Draught horse intact males) or MIX (Draught horse and Haflinger intact males) regarding farm size (total number of animals, number of stallions, mares and geldings), *T. equigenitalis*-positive tested animals, contact of the stallions to: stallions (S)/ geldings (G) / mares (M), contact with *T. equigenitalis* before (CEM before Y/N), *T. equigenitalis* test regime, other horse breeds on the same farm and age distribution of *T. equigenitalis*-positive intact males and total tested intact males; Y=yes, N=no, NA=not available. [1]There have been known cases of CEM-positive animal on the farm. [2]A regular CEM testing system before the breeding season was carried out on the farm. [3]Horses of breeds other than the breeds tested were housed on the farm.

**Farm No.**	**Farm size**		**Intact males**	**Contact of the stallions to:**	**CEM before[1]**	**CEM test regime[2]**	**Other breeds[3]**	**Age distribution Median (Min-Max)**	
	**Total number**	**Number of stallions/mares/geldings**	**(tested positive / total number tested)**	**S/G/M**	**Y/N**	**Y/N**	**Y/N**	**tested positive**	**tested total**
				**ICE**					
**ICE 1**	>100	>20/>20/>20	15/34	S/G/M	Y	Y	N	4 (3 – 12)	4 (3 – 23)
**ICE 2**	50-100	5-20/>20/5-20	0/4	S/M	Y	Y	N	-	11.5 (6 – 18)
**ICE 3**	<50	5-20/5-20/5-20	1/9	S/M/G	N	Y	N	6	5 (3 – 9)
**ICE 4**	NA	NA	0/1	NA	NA	NA	NA	-	11
**ICE 5**	NA	NA	0/1	NA	NA	NA	NA	-	3
**ICE 6**	<50	5-20/0/0	0/4	S	N	N	N	-	2.5 (1.5 – 5)
**ICE 7**	NA	NA	3/6	NA	NA	NA	NA	3 (3 – 4)	4.5 (3 – 16)
**ICE 8**	50-100	>20/>20/>20	0/6	S/G/M	N	Y	N	-	13 (6 – 26)
**ICE 9**	>100	5-20/>20/>20	4/10	S/G	N	Y	Y	1.5 (1.5 – 2.5)	1.5 (1.5 – 2.5)
**ICE 10**	50-100	<5/>20/>20	0/1	S/G/M	N	Y	N	-	16
				**HAF**					
**HAF 1**	50-100	>20/5-20/5-20	0/15	S/M	N	N	Y	-	4 (2.5 – 16)
**HAF 2**	<50	<5/>20/0	0/1	S/M	NA	NA	Y	-	6

**HAF 3**	NA	NA	0/1	NA	NA	NA	NA	-	5
**HAF 4**	<50	<5/NA/NA	0/2	S/M	N	N	N	-	5.5 (4 – 7)
**HAF 5**	<50	<5/NA/NA	0/1	S/M	N	N	N	-	13
**HAF 6**	<50	<5/NA/NA	0/1	S/M	N	N	N	-	4
**HAF 7**	<50	<5/NA/NA	0/1	S/M	N	N	N	-	6
**HAF 8**	<50	<5/NA/NA	0/2	S/M	N	N	N	-	9.5 (6 – 13)
**HAF 9**	<50	<5/NA/NA	0/2	S/M	N	N	N	-	9 (9 - 9)
**HAF 10**	<50	<5/NA/NA	0/2	S/M	N	N	Y	-	6 (5 – 7)
**HAF 11**	<50	<5/NA/NA	0/1	S/M	N	N	N	-	4
**HAF 12**	NA	NA	0/1	NA	NA	NA	NA	-	3
**Draught**									
**Draught 1**	<50	<5/5-20/5-20	0/3	S/G/M	N	Y	Y	-	2.5 (2.5 – 5)
**Draught 2**	NA	NA	0/4	NA	NA	NA	NA	-	3 (2.5 – 11)
**Draught 3**	50-100	<5/>20/>20	0/1	M	N	N	Y	-	11
**Draught 4**	NA	NA	0/1	NA	NA	NA	NA	-	4
**Draught 5**	<50	<5/5-20/5-20	0/1	S/G/M	N	Y	Y	-	5
**Draught 6**	<50	5-20/0/<5	0/8	S/G	N	N	N	-	4.5 (2 – 15)
**Draught 7**	<50	5-20/0/5-20	2/5	S/G	N	N	N	8.5 (7 – 10)	7 (3 – 10)
**Draught 8**	NA	NA	0/1	NA	NA	NA	NA	-	6
**Draught 9**	<50	<5/<5/<5	0/1	S/G/M	N	N	N	-	3
**Draught 10**	NA	NA	0/2	NA	NA	NA	NA	-	7 (7 – 7)
**Draught 11**	NA	NA	2/4	NA	NA	NA	NA	8 (8 - 8)	5.25 (2.5 – 8)
**Draught 12**	<50	5-20/5-20/5-20	0/3	S/G/M	N	N	Y	-	2.5 (2 – 3)
**Draught 13**	NA	NA	0/3	NA	NA	NA	NA	-	9 (4 – 16)
**Draught 14**	NA	NA	0/1	NA	NA	NA	NA	-	2
**MIX (HAF/Draught)**									
**MIX 1**	NA	NA	0/13	NA	NA	NA	NA	-	8 (4 – 16)
**MIX 2**	NA	NA	0/5	NA	NA	NA	NA	-	15 (14 – 18)
